# The impact of surface pre-reacted glass-ionomer nanoparticles on ion dynamics and the physio-mechanical properties of denture base resin

**DOI:** 10.1590/1678-7757-2025-0060

**Published:** 2025-10-20

**Authors:** Kwanchanok RATANAKUPT, Toshiyuki NAKATSUKA, Kritirat KIATSIRIROTE

**Affiliations:** 1 Srinakarinwirot University Faculty of Dentistry Department of Conservative Dentistry and Prosthodontics Bangkok Thailand Srinakarinwirot University, Faculty of Dentistry, Bangkok, Department of Conservative Dentistry and Prosthodontics, Thailand.; 2 Autonomous Researcher Kyoto Japan Autonomous Researcher, Kyoto, Japan.; 3 Thammasat University Faculty of Dentistry Prosthodontics Department Thailand Thammasat University, Faculty of Dentistry, Prosthodontics Department, Thailand.

**Keywords:** S-PRG filler, Nanoparticle, Denture base resin, Ion release, Bioactive glass

## Abstract

Dentures containing nanoparticles must maintain appropriate ion dynamics and physio-mechanical properties.

**Objective:**

this study evaluated the effects of incorporating 5wt% and 10wt% surface pre-reacted glass-ionomer S-PRG nanoparticles into a polymethylmethacrylate (PMMA) resin on multiple-ion exchange rates following recharge with an S-PRG solution, flexural strength, surface roughness, and hardness.

**Methodology:**

In total, 72 disc-shaped and 32 rectangular specimens were fabricated from PMMA resin containing 0, 5, and 10% S-PRG nanoparticles, and 20% S-PRG microparticles by weight. In total, six-disc specimens per group were tested for surface roughness and hardness using a contact profilometer and the Vickers hardness test, respectively. The remaining discs were immersed in 11 ml of deionized water for 24 hours, followed by an analysis of their aluminum, boron, sodium, silicate, strontium, and fluoride concentrations. They were then recharged with a 10% S-PRG solution for 24 hours, alternating with deionized water for five cycles, before their ion levels were reanalyzed. Rectangular specimens underwent flexural strength testing via the three-point bending method. Data were analyzed using two-way repeated-measures analysis of variance with a significance level of p<0.05.

**Results:**

PMMA resin modified with S-PRG nanoparticles released all ions except aluminum. The 10wt% nanoparticle group showed significantly higher ion release than the 5wt% nanoparticle and control groups. While the initial ion release from the 10wt% nanoparticle was comparable to the 20wt% microparticle, recharge resulted in slower ion release from nanoparticles. Incorporating S-PRG nanoparticles altered the surface characteristics and flexural strength of the resin (which remained within international standards).

**Conclusion:**

Incorporating 5 and 10wt% S-PRG nanoparticles into PMMA resin facilitated the release of boron, silicate, strontium, sodium, and fluoride ions, but not of aluminum ones following recharge with an S-PRG solution and maintained compliance with standards. The 10wt% nanoparticles achieved a balance between enhanced ion release and the physio-mechanical properties required for denture base resins.

## Introduction

With increasing life expectancy, older adults are more likely to experience partial or complete tooth loss, which can significantly impact nutrition and quality of life. Strategies for replacing lost teeth vary, but removable polymethylmethacrylate (PMMA) resin dentures remain the most common due to their cost, ease of use, ability to reparability, and aesthetic satisfaction.^1^However, a major drawback for patients using removable dentures refers to the increased risk of caries and periodontal disease, which complicates proper hygiene. Additionally, PMMA encourages microbial colonization and adhesion, leading to oral candidiasis. Clinical and epidemiological studies have established a strong correlation between high plaque scores and denture stomatitis.^2^

The antimicrobial denture concept provides an alternative solution for addressing oral candidiasis by incorporating antifungal agents into dental materials and enabling their controlled release.^3^ However, the release of antibiotics into the oral environment is often transient as these agents rapidly dissipate in saliva.^4^ This rapid dissolution reduces treatment effectiveness and may contribute to the development of microbial resistance to antibiotics.^5^ The application of various types of bioactive nanoparticles on antimicrobial denture has been proposed to mitigate microbial resistance based on their unique characteristics. Those include their large surface area-to-volume ratio, ability to deliver significant amounts of antibiotics, extensive interactions with microorganisms, and small particle sizes and shapes, which enhance microbial attachment and antimicrobial efficacy.^6,7^ Although the antimicrobial mechanisms of nanoparticles are yet to be fully understood, studies suggest they involve oxidative stress modulation, ion release, or other non-oxidative processes.^8,9^

Various inorganic nanoparticles, including those made from gold, ceramics, and metallic oxides, have been synthesized to combat microbial infections. These nanoparticles undergo gradual hydrolysis, exchanging ions with their environment based on factors such as size, shape, concentration, and interaction with the resin matrix.^10^ Among these, surface pre-reacted glass-ionomer (S-PRG) nanoparticles show particular promise due to their ability to release bioactive ions, including sodium (Na), borate (B), aluminum (Al), silicate (Si), strontium (Sr), and fluoride (F). These ions strengthen teeth, buffer acids, and inhibit demineralization and microbial formation.^11^ Studies have evinced that materials containing S-PRG show antibacterial activity against oral pathogens, particularly *Streptococcus mutans,* primarily by releasing borate and fluoride ions.^12^ Moreover, ion release from S-PRG fillers have been reported to suppress the progression of periodontal disease and inhibit the penetration of *Porphyromonas gingivalis*virulence factors into gingival epithelial cells.^13^ Tsutsumi, Takakuda, and Wakabayashi^14^ (2016) have also shown that eluates from S-PRG fillers prevent *Candida albicans*adhesion to resin-based materials by inhibiting the yeast-to-hyphae transition, a mechanism that may contribute to the prevention of denture stomatitis.

S-PRG nanoparticles have a unique structure with a SiO2 protection layer, a pre-reacted glass-ionomer phase, and a fluoro-boro-aluminosilicate glass core. This enables a dynamic ion exchange for long-term antimicrobial effects.^13^ These nanoparticles have been incorporated into denture base resins, tissue conditioners, and coating materials, and have shown their effectiveness in various applications.^11,15,16^ Studies have indicated that adding 20wt% S-PRG microparticles may compromise ion release efficacy and maintain acceptable mechanical strength in resin denture bases in accordance with ISO standards.^17,18^ However, our previous study has shown that incorporating 20wt% S-PRG nanoparticles into PMMA resin enhances fluoride release more effectively than microparticles at the same concentration.^16^ Despite this benefit, such a high nanoparticle loading reduces the flexural strength to below 65 MPa, thereby failing to meet the ISO 20795-1 requirements. The degree of ion release depends on resin composition, filler content, and interaction with the resin matrix. Therefore, while higher nanoparticle content can enhance ion release, it may compromise the physical properties of the material due to agglomeration.^15,16^

Thus, incorporating nanoparticles into resins require balancing ion exchange rates and physio-mechanical performance for clinical effectiveness. However, limited data exist on the incorporation of S-PRG nanoparticles into resin materials for multiple-ion release. This study incorporated 5 and 10wt% S-PRG nanoparticles into resin materials and evaluated the effects on the dynamic exchange of B, Al, Si, Sr, Na, and F ions following recharge with an S-PRG solution. Their impact on the flexural strength, surface roughness, and hardness of PMMA resin were also evaluated. The null hypothesis of this study posited that conventional resin and resin incorporated with S-PRG nanoparticle would show no differences regarding ion-exchange capability, mechanical properties, or surface characteristics.

## Methodology

### Sample preparation

In total, 72 disc-shaped (15**×**3 mm) and 32 rectangular (64**×**10**×**3 mm) specimens were prepared from PMMA resin (URBAN RESIN, Lot 081905, Shofu Inc., Kyoto, Japan) containing 0, 5, and 10wt% nanoparticles (average particle size: 400 nm or 0.4 µm) and 20wt% microparticles (average particle size: 3.0 µm) of S-PRG fillers. The filler particles were mechanically dispersed into the PMMA powder supplied by the manufacturer and mixed with methyl methacrylate monomer (Lot 041950, Shofu Inc., Kyoto, Japan) at a ratio of 10 g/5 ml, following the manufacturer’s instructions. The mixture was placed into a metal mold, compressed under a pressure of 6.1 MPa for 10 minutes, then immersed in a heat-retention water bath at 100°C for 45 minutes. After polymerization, the samples were removed from the mold and ground using 600-2,000-grit silicon carbide abrasive paper under dry conditions. The rectangular specimen dimensions (64**×**10**×**3 mm) were selected following ISO 20795-1 for the mechanical testing of denture base polymers. Similarly, the disc-shape specimens (15**×**3 mm) (commonly used in ion release studies)were prepared based on methodologies in theliterature.^14,16,17^ The dimensional accuracy of the samples was verified using a Vernier caliper (RS Pro Electronic Digital Caliper, RS Pro, Bangkok, Thailand) in accordance with ISO 20795-1 requirements.

### Elemental analysis

In total, 24 disc-shaped specimens (n=6/group) were stored in individual plastic containers with 11.0 ml of deionized water at 37°C for 24 hours over a 15-day period to determine the initial ion release from the materials. The storage solution was discarded and replaced daily with an equal volume of fresh deionized water. On days 1, 2, and 15, a 6-ml portion of the solution was analyzed for Al, B, Na, Si, and Sr ion concentrations using inductively coupled plasma atomic emission spectroscopy (ICP-AES; ICPS-8000, Shimadzu Co., Kyoto, Japan). Additionally, a 5-ml portion was used to determine fluoride concentrations with an ion-selective electrode connected directly to an ion meter (ISE, Model 9609BN, Orion Research; pH/ion meter: Model 720A, Orion Research) on the same days (1, 2, and 15).

The specimens were then processed for rechargeability by immersion in a 10% S-PRG solution for 24 hours. Following immersion, the specimens were rinsed and stored in deionized water for another 24 hours. The ion release rates after recharge were analyzed over five 10-day cycles, with measurements taken on days 17, 19, 21, 23, and 25 using the same analytical methods. Standard solutions containing multiple elements and sodium fluoride were prepared to calibrate the measurements. The 10% S-PRG solution was prepared by diluting ProCare Gel^®^ (containing 5wt% S-PRG filler, Lot 072101, Shofu Inc., Kyoto, Japan) in deionized water at a 1:10 ratio and resting it for 24 hours to ensure uniform ion distribution before immersing the specimens for recharge.

### Flexural strength

Rectangular specimens (n=8) were prepared and tested in accordance with ISO 20795-1. Specimens were stored in distilled water at room temperature for 50 hours before undergoing a three-point bending test at a crosshead speed of 5 mm/min using a universal testing machine (446 Material, Instron, Darmstadt, Germany). The load at fracture was recorded and used to calculate flexural strength using the formula: *σ =* 3Fl/2bh^2^, in which σ is the flexural strength (MPa), F is the load at fracture (N), l is the distance between the supporting span (mm), b is the width of the specimen (mm), and h is the height of the specimen (mm). The fractured surfaces of representative specimens from each group were examined, and their elemental spectra were analyzed using a scanning electron microscope with energy-dispersive X-ray spectroscopy (SEM/EDS, JSM-7800F; JEOL, Welwyn Garden City, England) at 1,000**×** and 10,000**×** magnifications.

### Surface roughness

In total, 24 disc-shaped specimens (n=6/group) were stored in distilled water for 72 hours before their surface roughness was measured using a contact profilometer (TALYScan 150, Taylor Hobson Ltd., Leicester, England). Overall, six surface areas of each specimen measuring 3**×**1 mm^2^ were randomly scanned to calculate average roughness (Ra), average maximum surface height (Rz), and maximum height (Rz max) in micrometers (µm). Specimens with Rz ≥ 1.0 µm or Rz max ≥ 1.0 µm were excluded from this study.

### Surface hardness

In total, 24 disc-shaped specimens (n=6/group) were stored in distilled water for 72 hours at room temperature before testing for Vickers hardness (HV) using a micro-hardness tester (FM-810, Future-Tech, Tokyo, Japan). A force of 300 g was applied to the specimen surfaces for 15 seconds. The lengths of the diagonals of the indentations were measured to calculate HV using the formula HV=F/A, in which F is the applied force in kilograms-force and A is the surface area of the indentation in mm^2^. Moreover, six indentations were made at random points on each specimen, the mean value of which was calculated for each sample.

### Statistical analysis

The sample size for each test was determined on G*Power, version 3.1.9.7, based on an effect size of 0.5 according to Cohen’s *d* and further adjusted with reference to a previous study.^14^ The calculations ensured a > 0.95 statistical power at an alpha level of 0.05. Data were analyzed on IBM SPSS, version 26, for macOS (SPSS Inc, Chicago, IL, USA). Normality was assessed using the Shapiro-Wilk test and homogeneity of variances was evaluated by the Levene’s test for each dependent variable. Differences in mean ion concentrations (Al, B, Na, Si, Sr, and F) between study groups were assessed using two-way repeated measured analysis of variance. Data for flexural strength (MPa), surface roughness (Ra, Rz, Rz max, µm), and surface hardness (HV) were compared between groups by one-way analysis of variance followed by the Tukey’s HSD *post hoc* test for multiple comparisons. All mean differences are shown with 95% confidence intervals, and a *p<0.05* was considered statistically significant.

## Results

### Ion release

**Figure 1:**
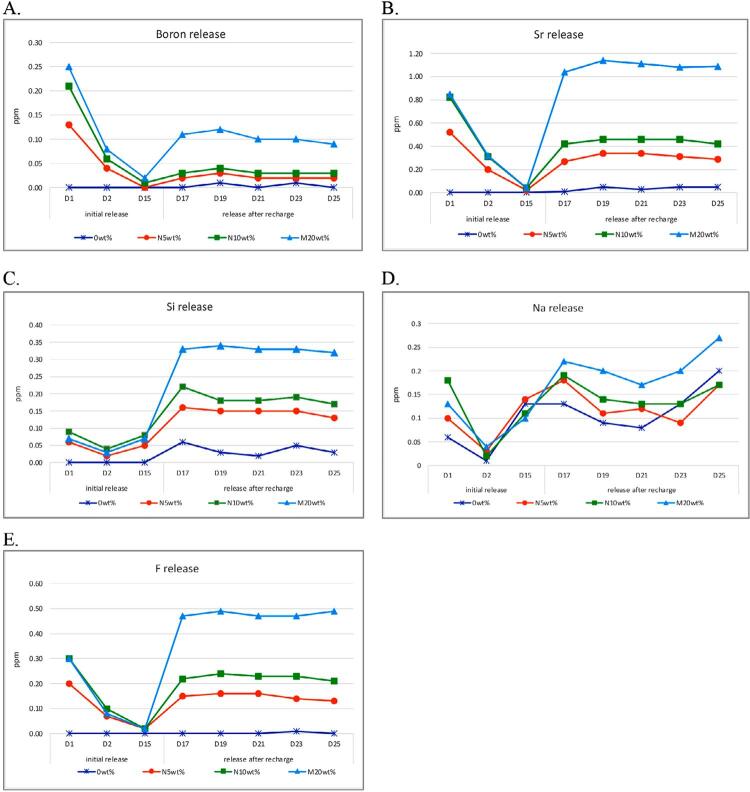
Trends in the release and recharge of Boron (A), Strontium (B), Silicon (C), Sodium (D), and Fluoride (E) Ion from PMMA resins at measured time points.

Concentrations of B, Si, Sr, Na, and F ions were detected in the resins containing S-PRG micro- and nanoparticles during the initial and recharge periods, whereas Al ion concentrations remained below 0.01 ppm (*i.e.*, undetectable) in all groups. The ion concentrations and release profiles of the experimental resin materials at measured time points are shown as mean values (ppm) in Figure 1.

The initial ion release rate from the resin containing 10wt% S-PRG nanoparticle was approximately equivalent to that of the 20wt% microparticles and significantly higher than the rates in the 5wt% nanoparticle and control resin without a S-PRG filler. Of all S-PRG fillers, Sr ions showed the highest concentration, followed by F, B, Na, and Si. Resins with S-PRG fillers showed similar release profiles due to the glass-ionomer base material, characterized by a rapid release on the first day, followed by a significant decrease on the second day. Subsequently, the release rates of B, Sr, and F ions gradually declined to below 0.04 ppm by day 15, whereas Si and Na ions showed release levels comparable to those observed on day 1.

Upon applying a 10% S-PRG solution for recharge, resins with S-PRG micro- and nanoparticles showed **a** significant increase in ion release rates, particularly during the first recharge cycle. Release rates remained consistent over five recharge cycles in all S-PRG groups. During recharge, the resin with 20wt% microparticles absorbed and released significantly higher levels of B, Si, Sr, Na, and F ions than the resins with 5 and 10wt% nanoparticles. Of all ions, Sr showed the highest release rate after repeated recharges, whereas B, the lowest. Boron release levels were measured at 0.02, 0.03, and 0.12 ppm in resins containing 5 and 10wt% nanoparticles and 20wt% microparticles, respectively.

The control resin without S-PRG fillers released only Na ions during the initial phase. However, it absorbed B, Si, Sr, and F ions from the recharged solution, albeit at a lower rate than the resins with S-PRG fillers. The Na ion release profile of the control resin after recharge was resembled that of resins containing 5 and 10wt% S-PRG nanoparticles.

### Flexural strength

Resin flexural strength, surface roughness, and hardness data are shown in Table 1. The control resin showed the highest flexural strength, significantly higher than that of the resins containing 5 and 10wt% S-PRG nanoparticles and 20wt% microparticles. Flexural strength decreased with increasing S-PRG filler content. Notably, the flexural strength of the resin with 5wt% nanoparticles significantly exceeded that of the resin with 10wt% nanoparticles. However, no significant difference was observed between the 10wt% nanoparticle and 20wt% microparticle groups. All resins showed flexural strength values exceeding 65 MPa, meeting the required standard.

**Table 1:** Mean (standard deviation) of flexural strength, surface roughness, and hardness of the PMMA resin.

Group	Flexural strength	Vickers hardness	Surface roughness (µm)		
	(MPa)	(HV)	Ra	Rz	Rzmax
0wt%	93.57(6.74)^a^	19.24(0.59)^a^	0.04(0.02)^a^	0.24(0.12)^a^	0.64(0.24)^a^
N5wt%	77.97(9.11)^b^	19.16(0.56)^a^	0.05(0.02)^a^	0.50(0.23)^a^	0.55(0.23)^a^
N10wt%	69.76(5.05)^c^	19.63(0.97)^a^	0.06(0.01)^a^	0.34(0.04)^a^	0.79(0.11)^a^
M20wt%	67.05(2.62)^c^	20.16(1.04)^a^	0.07(0.01)^a^	0.37(0.11)^a^	0.81(0.26)^a^

Group identified by different superscript letters showed significant differences (p>0.05).

### Surface roughness and hardness

Surface roughness and hardness of denture bases are critical factors influencing microbial colonization. Roughness measurements (Ra, Rz, and Rz max, µm) and HV values increased with higher S-PRG filler content and particle size. However, the roughness values of all experimental resins remained below the international standard (0.2 µm). The HV of the resin with microparticles remained slightly higher than that of the control resin and the resins with nanoparticles, although with no statistically significant differences.

### SEM analysis

**Figure 2:**
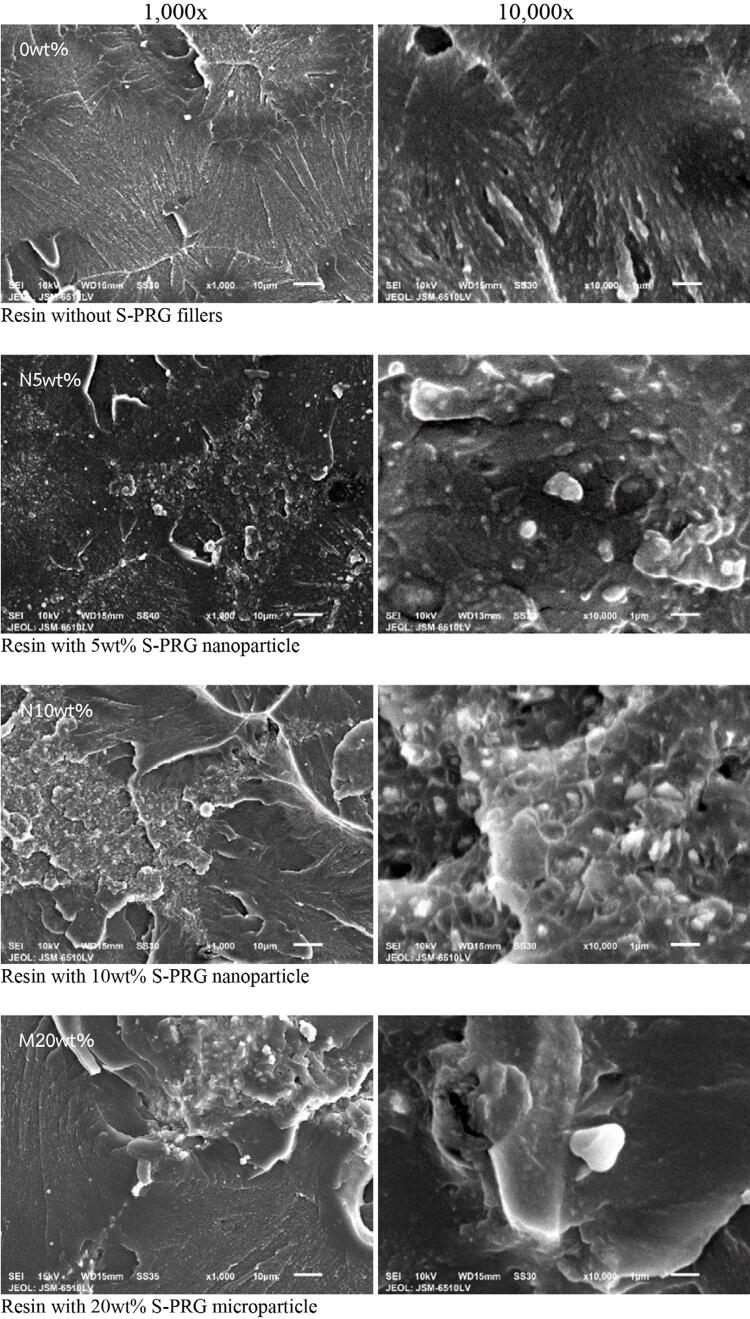
Fractured surfaces of the tested specimens at 1,000- and 10,000-× magnification.

**Figure 3:**
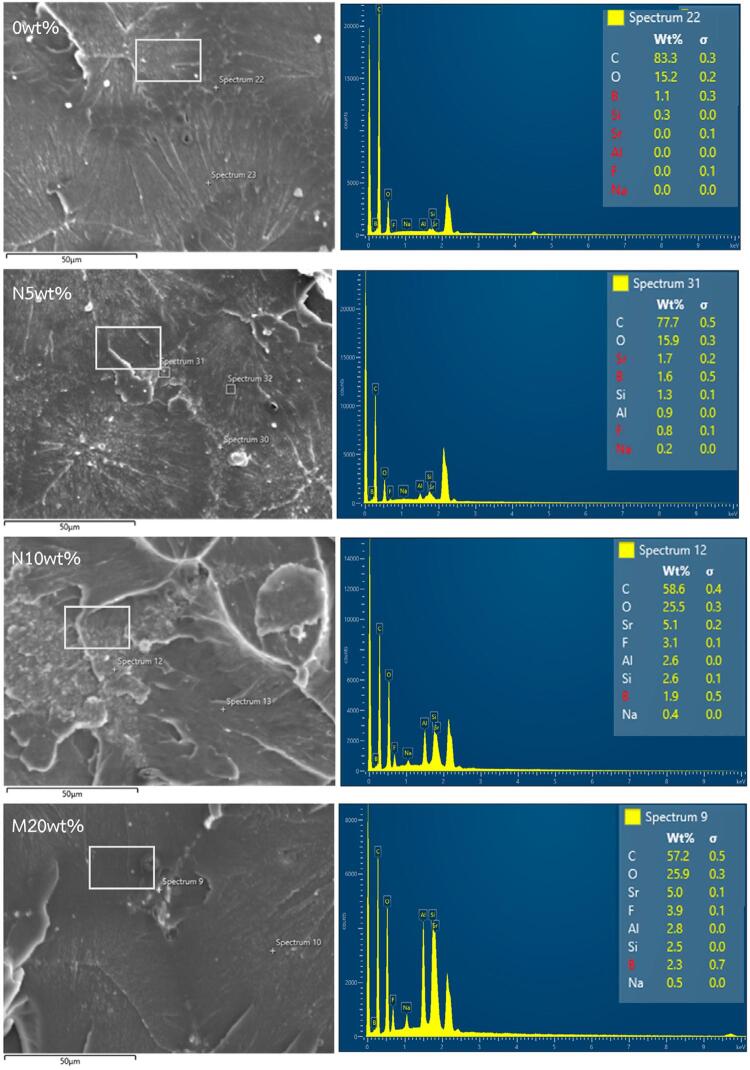
EDS images and detected elements from the tested specimens.

SEM images and the EDS spectra of the fractured surfaces after the three-point bending test are shown in Figure 2 and 3. Resins containing nano- and micro-sized S-PRG fillers showed radiopaque particle distribution in their matrix, whereas the control, a smooth particle-free surface. Smaller nanoparticles were well-distributed in the matrix, although agglomeration was observed in the 5 and 10wt% groups, appearing as larger particles than the microparticles. However, localized agglomeration was evident in the 5 and 10wt% nanoparticle groups, appearing as larger composites cluster along the fracture surface of the modified resins. These clusters likely resulted from the high surface energy of nanoparticles and their tendency to aggregate during mixing and polymerization. EDS analysis confirmed the presence of B, Si, Sr, F, Na, and Al in the S-PRG filler particles. Of these, Sr ions showed the highest spectral intensity across all S-PRG resins, consistent with their ion release behavior. Al ions, although a component of the S-PRG filler, were undetectable in the released solution. The loss of Al ion exchange capability may result from resin polymerization or the stable structure of cationic exchangers.

## Discussion

Incorporating nanoparticles to enhance the biological properties of denture base resins has been extensively studied due to their unique characteristics, including small size, shape, and reactivity.^3,6^ S-PRG nanoparticles have showed their ability to facilitate dynamic ion exchange (B, Al, Si, Sr, Na, and F) and are expected to improve the physio-mechanical properties of resins when compared to microparticles.^11,15^ The null hypothesis of this study is partially rejected based on its results. This study confirmed that ion release from S-PRG nanoparticles depends on filler content. As with previous studies, the release pattern showed a burst effect and gradual decrease over time for B, Sr, and F ions.^15,16^ This behavior, characteristic of resin-based materials containing glass fillers, reflects the buffering capacity of ions released from S-PRG fillers. These fillers gradually deplete stored ions to neutralize acids, consistently reducing pH levels in storage solutions.^19,20^ The acid-neutralizing capacity of S-PRG fillers has been reported to inhibit bovine enamel demineralization, induce oxidative stress, and prevent *C. albicans*from transitioning to its hyphal form during early contact.^14,18,21^Additionally, the solubility and water sorption properties of PMMA resin facilitated ion exchange from the S-PRG filler; beginning with the dissolution of surface-bound ions, followed by the gradual removal of residual monomers within the resin matrix. This process created spaces that enabled potential ion re-exchange driven by concentration gradients.^22,23^

Notably, the resin incorporating 10wt% S-PRG nanoparticles showed an initial release rate comparable to that of a 20wt% microparticle resin. The smaller particle size, larger surface area, and stronger interfacial adhesion of nanoparticles likely enhanced their attachment to the resin surface, facilitating water penetration and enabling the high initial ion release rates in this study.^15,16^ These findings agree with previous studies showing that S-PRG nanoparticles significantly enhanced the fluoride-releasing capability of resin base materials when compared to microparticles.^13,16^ The high initial ion release levels from nanoparticles may offer greater clinical benefits for S-PRG over microparticles.^19,21^Supporting this, Mayumi, et al.^11^(2021) reported that 0.48-µm S-PRG nanoparticles firmly adhere to dentin surfaces and show bioadhesive properties that enhance antibacterial efficacy, whereas microparticles were easily removed.

The morphological characteristics of S-PRG have been documented. A prior SEM study reported that nano- and microparticles of pure S-PRG show irregular polygonal microstructures, with nanoparticles being smaller and more homogeneous in size.^15^ Our earlier investigation using dynamic light scattering analysis confirmed that S-PRG nanoparticles incorporated into PMMA resin had an average particle size of 386.53±0.83 nm (approximately 0.38 µm), consistent with the manufacturer’s specifications.^16^ Given this prior characterization, detailed morphological and particle size analyses were ignored in this study.

Given the gradual decrease in ion levels over time, an ideal denture material should be rechargeable to maintain consistent ion levels and support long-term therapeutic functions.^17^ This concept is in line with common patient routines, in which denture wearers are advised to maintain daily oral hygiene and store their dentures overnight in water. Various recharging protocols, including the regular use of fluoride toothpastes or fluoride solutions, have been used to release fluoride, which effectively strengthens enamel.^24^ Studies have shown a dynamic pattern of delayed fluoride release in dentures containing S-PRG fillers under regular fluoride recharge regimens.^17,25^ However, most fluoride toothpastes or solutions are limited to single fluoride applications.

To address this limitation, this study proposed an alternative recharge protocol using a 10% S-PRG solution as an external ion source to replenish bioactive components in denture base resins. Following this protocol, all resins containing S-PRG fillers showed significant ion uptake and subsequent release with consistent performance across five recharge cycles. Interestingly, the enhanced ion exchange in the 10wt% nanoparticle group during the initial phase was absent during recharge. This may be due to fewer and smaller perforations created by nanoparticles within the resin matrix, which limit water absorption and ion diffusion when compared to the large perforation associated with microparticles, as previously illustrated by SEM imaging.^16^ Although the 20wt% microparticle resins showed the greatest overall ion release attributable to its greater filler content, the 10wt% nanoparticle resin maintained effective rechargeability while offering a more favorable balance between ion release and flexural strength.

In terms of ion-specific release, Sr consistently showed the highest release level in the initial and recharge phases, followed by F, B, Na, and Si. This pattern is in line with previous findings for S-PRG containing materials and reflects the relative abundance and solubility of these ions within the fluoro-boro-aluminosilicate glass core.^19,26^ Strontium is particularly notable for its remineralizing effects, ability to enhance osteoblastic activity, and antibacterial properties, suggesting that adding S-PRG to resins may benefit hard- and soft-tissue interfaces.^12,27^Moreover, even low concentrations of borate have been shown to inhibit the growth of *Candida albicans* and *Streptococcus mutans*by destabilizing cell membranes and suppressing cell metabolism.^14,28,29^

Na ions showed a distinct release pattern, with levels rising after each recharge cycle for all groups. Na ions contribute to the high solubility and water absorption of PMMA resins, and its rapid washout triggers the exchange of other ions to maintain electroneutrality.^30-32^ Interestingly, the conventional resin released Na ions at levels comparable to those of the modified resin containing S-PRG fillers despite its lower rechargeability for other ions. This higher Na release in the conventional resin may result from free perforations being replaced with water, enhancing sorption. However, prolonged water exposure compromises the structural integrity and volumetric stability of PMMA, reducing strength and causing degradation.^33,34^

Al release has been associated with reduced tooth hypersensitivity. However, Al ions were undetectable in the eluates, as in previous findings on S-PRG-containing tissue conditioners.^15^ The absence of Al ion released solution may be attributed to its incorporation into chemicallystable sites within the glass network or its immobilization during resin polymerization.^35^The factors for variations in ion release behaviors remain unclear but may be influenced by ionic radius, chemical interactions within the glass-ionomer phase, the solubility of the modified resin-based materials, among others.^15,29^

The cytocompatibility of S-PRG fillers has been studied. Nemoto, et al.^29^ (2018) have reported that eluates from S-PRG upregulated osteogenic gene expression in human bone marrow-derived stromal cells without inducing cytotoxicity, promoting extracellular matrix mineralization. Similarly, Yang, et al.^36^ (2025) reported enhanced adhesion, proliferation, and osteogenic differentiation of human periodontal ligament stem cells cultured on S-PRG-containing resins. Furthermore, S-PRG stimulated cell migration and capillary-like tube formation indicating its potential to support angiogenesis *in vivo*. These studies suggest that incorporating bioactive S-PRG particles into resin materials enhances their biocompatibility and osteogenic potential, offering a promising alternative for clinical applications.

While the ion exchange capabilities were assessed for their potential to prolong long-term antimicrobial properties, flexural strength remains critical for functional performance. Previous studies have shown that increasing filler content and decreasing filler size can weaken the mechanical properties of materials.^22,23,37^ S-PRG nano- and microparticles were incorporated into a PMMA resin without undergoing chemical reactions, preserving filler bioactivity. However, the high reactivity of nanoparticles caused agglomeration, especially at higher concentrations, leading to a significantly lower flexural strength in the 10wt% nanoparticle group when compared to the 5wt% and conventional resin groups. Moreover, SEM images of the fractured surface showed a uniform distribution of nanoparticles within the resin despite localized agglomeration in both nanoparticle groups, appearing as clusters of larger composite domains. These agglomerates likely contributed to the reduction of flexural strength in this study. Importantly, all flexural strength values exceeded the 65-MPa requirement of the international standard. However, the reduced flexural strength at 10wt% nanoparticle loading narrows their mechanical safety margin, particularly in areas susceptible to stress concentration and fracture. Patients with high functional loading may require increasing the bulk thickness in stress-bearing areas and appropriate prosthesis design and maintenance to ensure long-term durability.

Surface roughness and hardness influence microbial colonization, color changes in denture base resin, wear resistance, and oral hygiene during denture use.^38^ Our findings indicated that incorporating S-PRG nanoparticles or microparticles gradually increased surface hardness (or the roughness parameters Ra, Rz, Rzmax) when compared to the conventional resin. However, these differences were statistically insignificant in the groups. Despite a gradual increase in surface roughness due to S-PRG filler incorporation, studies have suggested a potential reduction in the growth and metabolic activity of *C. albicans*, decreasing the risk of denture stomatitis.^14,15^ Notably, the roughness average of all experimental resins remained within the acceptable limit of 0.2 µm, above which plaque accumulation is likely to increase.^14^ Furthermore, the observed increase in HV may be attributed to the uniform distribution of S-PRG nanoparticles on the specimen surface and the higher filler content of microparticles within the resin matrix.^37^ Similar findings regarding surface hardness and roughness have been reported when inorganic oxide nanoparticles, such as zirconium, titanium, or silicon dioxide, were incorporated into PMMA resin.^39^

Various bioactive glass fillers have been explored for incorporation into dental resins, each with distinct ion-release characteristics. A BioUnion filler composed of SiO₂, ZnO, CaO, and F⁻ releases Zn^2^⁺, Ca^2^⁺, and F⁻. Zn^2^⁺ is particularly effective against *Streptococcus mutans*, with lower minimum inhibitory and bactericidal concentration values than fluoride.^12^ Its pH-responsive release increases under acidic conditions, enabling targeted on-demand antimicrobial action. Bioglass 45S5 and 65S, consisting of SiO₂, CaO, and P₂O₅ (± Na₂O), release Ca^2^⁺ and phosphate ions that raise local pH, inhibiting bacterial growth and reducing marginal infiltration. However, their antimicrobial effects are typically short-lived and require high filler concentrations, which may compromise the mechanical integrity of denture base resins.^22,23^ In contrast, S-PRG fillers provide sustained release and the ability to recharge multiple ions, furthering its potential for long-term effectiveness. These characteristics make S-PRG particularly advantageous for denture base applications, regarding which prolonged exposure to oral environment and microbial colonization are common concerns.

While surface roughness and hardness were unaffected by the addition of S-PRG and flexural strength complied with ISO 20795-1, this study ignored other ISO-mandated properties such as water sorption, solubility, or impact strength. Therefore, some ISO standards remain to be confirmed. Finally, while this study neither evaluated the long-term antimicrobial efficacy and cytotoxicity of S-PRG nanoparticles nor stimulated complex oral conditions such as thermal variations or pH fluctuations, future research should address these limitations to better understand the clinical potential of S-PRG-modified denture materials.

## Conclusion

This study showed that incorporating 5 and 10wt% S-PRG nanoparticles into PMMA resin materials enables measurable ion release and rechargeability, particularly for Sr, F, and B ions. The 10wt% nanoparticle group showed ion release levels comparable to those of 20wt% S PRG microparticles. Although increased filler content reduces flexural strength and slightly increases surface roughness and hardness, all values remained within clinically acceptable limits. These findings support the strategic incorporation of S-PRG nanoparticles to enhance the therapeutic potential of PMMA-base dental materials and provide long-term, rechargeable bioactivity.

## References

[1] 1 - Preshaw PM, Walls AW, Jakubovics NS, Moynihan PJ, Jepson NJ, Loewy Z. Association of removable partial denture use with oral and systemic health. J Dent. 2011;39(11):711-9. doi: 10.1016/j.jdent.2011.08.018 10.1016/j.jdent.2011.08.018 21924317

[2] 2 - Zafar MS. Prosthodontic applications of polymethyl methacrylate (PMMA): an update. Polymers. 2020;12(10):2299. doi: 10.3390/polym12102299 10.3390/polym12102299 PMC7599472 33049984

[3] 3 - Sharmin S, Rahaman MM, Sarkar C, Atolani O, Islam MT, Adeyemi OS. Nanoparticles as antimicrobial and antiviral agents: a literature-based perspective study. Heliyon. 2021;7(3):e06456. doi: 10.1016/j.heliyon.2021.e06456 10.1016/j.heliyon.2021.e06456 PMC7973307 33763612

[4] 4 - Lee JH, El-Fiqi A, Jo JK, Kim DA, Kim SC, Jun SK, et al. Development of long-term antimicrobial poly(methyl methacrylate) by incorporating mesoporous silica nanocarriers. Dent Mater. 2016;32(12):1564-74. doi: 10.1016/j.dental.2016.09.001 10.1016/j.dental.2016.09.001 27671462

[5] 5 - Swamy K, Alla RK, Mohammed S, Konakanchi A. The role of antifungal agents in treating denture stomatitis. Research J Pharm Tech. 2018;11(4):1365-19. doi: 10.5958/0974-360X.2018.00254

[6] 6 - Saini RS, Bavabeedu SS, Quadri SA, Gurumurthy V, Kanji MA, Okshah A, et al. Mapping the research landscape of nanoparticles and their use in denture base resins: a bibliometric analysis. Discov Nano. 2024;19(1):95. doi: 10.1186/s11671-024-04037-1 10.1186/s11671-024-04037-1 PMC11139848 38814562

[7] 7 - Cha SH, Hong J, McGuffie M, Yeom B, VanEpps JS, Kotov NA. Shape-dependent biomimetic inhibition of enzyme by nanoparticles and their antibacterial activity. ACS Nano. 2015;9(9):9097-105. doi: 10.1021/acsnano.5b03247 10.1021/acsnano.5b03247 26325486

[8] 8 - Abualsaud R, Gad MM. Highlights on drug and ion release and recharge capacity of antimicrobial removable prostheses. Eur J Dent. 2023;17(4):1000-11. doi: 10.1055/s-0042-1758788 10.1055/s-0042-1758788 PMC10756732 36574783

[9] 9 - Bacali C, Baldea I, Moldovan M, Carpa R, Olteanu DE, Filip GA, et al. Flexural strength, biocompatibility, and antimicrobial activity of a polymethyl methacrylate denture resin enhanced with graphene and silver nanoparticles. Clin Oral Investig. 2020;24(8):2713-25. doi: 10.1007/s00784-019-03133-2 10.1007/s00784-019-03133-2 31734793

[10] 10 - An S, Evans JL, Hamlet S, Love RM. Incorporation of antimicrobial agents in denture base resin: a systematic review. J Prosthet Dent. 2021;126(2):188-95. doi: 10.1016/j.prosdent.2020.03.033 10.1016/j.prosdent.2020.03.033 32800329

[11] 11 - Mayumi K, Miyaji H, Miyata S, Nishida E, Furihata T, Kanemoto Y, et al. Antibacterial coating of tooth surface with ion-releasing pre-reacted glass-ionomer (S-PRG) nanofillers. Heliyon. 2021;7(2):e06147. doi: 10.1016/j.heliyon.2021.e06147 10.1016/j.heliyon.2021.e06147 PMC7889979 33644453

[12] 12 - Imazato S, Kohno T, Tsuboi R, Thongthai P, Xu HH, Kitagawa H. Cutting-edge filler technologies to release bio-active components for restorative and preventive dentistry. Dent Mater J. 2020;39(1):69-79. doi: 10.4012/dmj.2019-350 10.4012/dmj.2019-350 31932551

[13] 13 - Imazato S, Nakatsuka T, Kitagawa H, Sasaki JI, Yamaguchi S, Ito S, et al. Multiple-ion releasing bioactive surface pre-reacted glass-ionomer (S-PRG) filler: Innovative technology for dental treatment and care. J Funct Biomater. 2023;14(4):236. doi: 10.3390/jfb14040236 10.3390/jfb14040236 PMC10142353 37103326

[14] 14 - Tsutsumi C, Takakuda K, Wakabayashi N. Reduction of Candida biofilm adhesion by incorporation of prereacted glass ionomer filler in denture base resin. J Dent. 2016 Jan;44:37-43. doi: 10.1016/j.jdent.2015.11.010 10.1016/j.jdent.2015.11.010 26655872

[15] 15 - Tonprasong W, Inokoshi M, Tamura M, Uo M, Wada T, Takahashi R, et al. Tissue conditioner incorporating a nano-sized surface pre-reacted glass-ionomer (S-PRG) filler. Materials (Basel). 2021;14(21):6648. doi: 10.3390/ma14216648 10.3390/ma14216648 PMC8588282 34772173

[16] 16 - Jitaluk P, Ratanakupt K, Kiatsirirote K. Effect of surface prereacted glass ionomer nanofillers on fluoride release, flexural strength, and surface characteristics of polymethylmethacrylate resin. J Esthet Restor Dent. 2022;34(8):1272-81. doi: 10.1111/jerd.12964 10.1111/jerd.12964 36169158

[17] 17 - Kamijo K, Mukai Y, Tominaga T, Iwaya I, Fujino F, Hirata Y, et al. Fluoride release and recharge characteristics of denture base resins containing surface pre-reacted glass-ionomer filler. Dent Mater J. 2009;28(2):227-33. doi: 10.4012/dmj.28.227 10.4012/dmj.28.227 19496404

[18] 18 - Mukai Y, Kamijo K, Fujino F, Hirata Y, Teranaka T, ten Cate JM. Effect of denture base-resin with prereacted glass-ionomer filler on dentin demineralization. Eur J Oral Sci. 2009;117(6):750-4. doi: 10.1111/j.1600-0722.2009.00678.x 10.1111/j.1600-0722.2009.00678.x 20121940

[19] 19 - Kaga N, Kaga M, Morita S, Nagano-Takebe F, Nezu T, Endo K, et al. Bioactive self-polymerizing resin with surface pre-reacted glass ionomer fillers for suppressed enamel demineralization. Materials (Basel). 2024;17(20):5101. doi: 10.3390/ma17205101 10.3390/ma17205101 PMC11509201 39459806

[20] 20 - Fujimoto Y, Iwasa M, Murayama R, Miyazaki M, Nagafuji A, Nakatsuka T. Detection of ions released from S-PRG fillers and their modulation effect. Dent Mater J. 2010;29(4):392-7. doi: 10.4012/dmj.2010-015 10.4012/dmj.2010-015 20610878

[21] 21 - Tamura M, Cueno ME, Abe K, Kamio N, Ochiai K, Imai K. Ions released from a S-PRG filler induces oxidative stress in Candida albicans inhibiting its growth and pathogenicity. Cell Stress Chaperones. 2018;23(6):1337-43. doi: 10.1007/s12192-018-0922-1 10.1007/s12192-018-0922-1 PMC6237688 29876727

[22] 22 - Raszewski Z, Chojnacka K, Mikulewicz M. Preparation and characterization of acrylic resins with bioactive glasses. Sci Rep. 2022;12(1):16624. doi: 10.1038/s41598-022-20840-1 10.1038/s41598-022-20840-1 PMC9534886 36198737

[23] 23 - Raszewski Z, Chojnacka K, Mikulewicz M, Alhotan A. Bioactive glass-enhanced resins: a new denture base material. Materials (Basel). 2023;16(12):46363. doi: 10.3390/ma16124363 10.3390/ma16124363 PMC10304369 37374547

[24] 24 - Lynch RJ, Navada R, Walia R. Low-levels of fluoride in plaque and saliva and their effects on the demineralisation and remineralisation of enamel; role of fluoride toothpastes. Int Dent J. 2004;54(5 Suppl 1):304-9. doi: 10.1111/j.1875-595x.2004.tb00003.x 10.1111/j.1875-595x.2004.tb00003.x 15509081

[25] 25 - Kiatsirirote K, Sitthisettapong T, Phantumvanit P, Chan DC. Fluoride-releasing effect of a modified resin denture containing S-PRG fillers on salivary fluoride retention: a randomized clinical study. Caries Res. 2019;53(2):137-44. doi: 10.1159/000490627 10.1159/000490627 PMC6348142 30056451

[26] 26 - Hatano K, Inokoshi M, Tamura M, Uo M, Shimizubata M, Tonprasong W, et al. Novel antimicrobial denture adhesive containing S-PRG filler. Dent Mater J. 2021;40(6):1365-72. doi: 10.4012/dmj.2020-443 10.4012/dmj.2020-443 34234047

[27] 27 - Thuy TT, Nakagaki H, Kato K, Hung PA, Inukai J, Tsuboi S, et al. Effect of strontium in combination with fluoride on enamel remineralization in vitro. Arch Oral Biol. 2008;53(11):1017-22. doi: 10.1016/j.archoralbio.2008.06.005 10.1016/j.archoralbio.2008.06.005 18672228

[28] 28 - Kitagawa H, Miki-Oka S, Mayanagi G, Abiko Y, Takahashi N, Imazato S. Inhibitory effect of resin composite containing S-PRG filler on Streptococcus mutans glucose metabolism. J Dent. 2018;70:92-6. doi: 10.1016/j.jdent.2017.12.017 10.1016/j.jdent.2017.12.017 29294301

[29] 29 - Nemoto A, Chosa N, Kyakumoto S, Yokota S, Kamo M, Noda M, et al. Water-soluble factors eluated from surface pre-reacted glass-ionomer filler promote osteoblastic differentiation of human mesenchymal stem cells. Mol Med Rep. 2018;17(3):3448-54. doi: 10.3892/mmr.2017.8287 10.3892/mmr.2017.8287 PMC5802126 29257332

[30] 30 - Chen X, Hill R, Karpukhina N. Chlorapatite glass-ceramics. Int J Appl Glass Sci. 2014;5(3):207-16. doi: 10.1111/ijag.12082

[31] 31 - Ogawa A, Wada T, Mori Y, Uo M. Time dependence of multi-ion absorption into human enamel from surface prereacted glass-ionomer (S-PRG) filler eluate. Dent Mater J. 2019;38(5):707-12. doi: 10.4012/dmj.2018-314 10.4012/dmj.2018-314 31189797

[32] 32 - Khvostenko D, Mitchell JC, Hilton TJ, Ferracane JL, Kruzic JJ. Mechanical performance of novel bioactive glass containing dental restorative composites. Dent Mater. 2013;29(11):1139-48. doi: 10.1016/j.dental.2013.08.207 10.1016/j.dental.2013.08.207 PMC3868470 24050766

[33] 33 - Alhotan A, Yates JM, Zidan S, Haider J, Jurado CA, Silikas N. Behaviour of PMMA resin composites incorporated with nanoparticles or fibre following prolonged water storage. Nanomaterials (Basel). 2021;11(12):3453. doi: 10.3390/nano11123453 10.3390/nano11123453 PMC8707186 34947803

[34] 34 - Dimitrova M, Vlahova A, Hristov I, Kazakova R, Chuchulska B, Kazakov S, et al. Evaluation of water sorption and solubility of 3D-printed, CAD/CAM milled, and PMMA denture base materials subjected to artificial aging. J Compos Sci. 2023;7(8):339. doi: 10.3390/jcs7080339

[35] 35 - Aydin N, Karaoglanoglu S, Aybala-Oktay E, Çetinkaya S, Erdem O. Investigation of water sorption and aluminum releases from high viscosity and resin modified glass ionomer. J Clin Exp Dent. 2020;12(9):e844-e51. doi: 10.4317/jced.56381 10.4317/jced.56381 PMC7511048 32994873

[36] 36 - Yang Y, Huang J, Hu X, Jing M, Zhang Y, Xu C, et al. Surface prereacted glass-ionomer particles incorporated into resin composites promote biocompatibility for restoration of subgingival dental defects. Mater Today Bio. 2025;31:101499. doi: 10.1016/j.mtbio.2025.101499 10.1016/j.mtbio.2025.101499 PMC11803238 39925721

[37] 37 - Kaga N, Morita S, Yamaguchi Y, Matsuura T. Effect of particle sizes and contents of surface pre-reacted glass ionomer filler on mechanical properties of auto-polymerizing resin. Dent J (Basel). 2023;11(3):72. doi: 10.3390/dj11030072 10.3390/dj11030072 PMC10047318 36975569

[38] 38 - Gad MM, Rahoma A, Al-Thobity AM. Effect of polymerization technique and glass fiber addition on the surface roughness and hardness of PMMA denture base material. Dent Mater J. 2018;37(5):746-53. doi: 10.4012/dmj.2017-191 10.4012/dmj.2017-191 29925729

[39] 39 - Azmy E, Al-Kholy MR, Al-Thobity AM, Gad MM, Helal MA. Comparative effect of incorporation of ZrO_2_, TiO_2_, and SiO_2_ nanoparticles on the strength and surface properties of PMMA denture base material: an in vitro study. Int J Biomater. 2022;2022:5856545. doi: 10.1155/2022/5856545 10.1155/2022/5856545 PMC9072016 35528846

